# Simulated space radiation sensitizes bone but not muscle to the catabolic effects of mechanical unloading

**DOI:** 10.1371/journal.pone.0182403

**Published:** 2017-08-02

**Authors:** Andrew R. Krause, Toni L. Speacht, Yue Zhang, Charles H. Lang, Henry J. Donahue

**Affiliations:** 1 Department of Orthopaedics, Penn State College of Medicine, Hershey, Pennsylvania, United States of America; 2 Department of Biomedical Engineering, Virginia Commonwealth University School of Engineering, Richmond, Virginia, United States of America; 3 Department of Cellular and Molecular Physiology, Penn State College of Medicine, Hershey, Pennsylvania, United States of America; Charles P. Darby Children's Research Institute, UNITED STATES

## Abstract

Deep space travel exposes astronauts to extended periods of space radiation and mechanical unloading, both of which may induce significant muscle and bone loss. Astronauts are exposed to space radiation from solar particle events (SPE) and background radiation referred to as galactic cosmic radiation (GCR). To explore interactions between skeletal muscle and bone under these conditions, we hypothesized that decreased mechanical load, as in the microgravity of space, would lead to increased susceptibility to space radiation-induced bone and muscle loss. We evaluated changes in bone and muscle of mice exposed to hind limb suspension (HLS) unloading alone or in addition to proton and high (H) atomic number (Z) and energy (E) (HZE) (^16^O) radiation. Adult male C57Bl/6J mice were randomly assigned to six groups: No radiation ± HLS, 50 cGy proton radiation ± HLS, and 50 cGy proton radiation + 10 cGy ^16^O radiation ± HLS. Radiation alone did not induce bone or muscle loss, whereas HLS alone resulted in both bone and muscle loss. Absolute trabecular and cortical bone volume fraction (BV/TV) was decreased 24% and 6% in HLS-no radiation vs the normally loaded no-radiation group. Trabecular thickness and mineral density also decreased with HLS. For some outcomes, such as BV/TV, trabecular number and tissue mineral density, additional bone loss was observed in the HLS+proton+HZE radiation group compared to HLS alone. In contrast, whereas HLS alone decreased muscle mass (19% gastrocnemius, 35% quadriceps), protein synthesis, and increased proteasome activity, radiation did not exacerbate these catabolic outcomes. Our results suggest that combining simulated space radiation with HLS results in additional bone loss that may not be experienced by muscle.

## Introduction

Deep space travel, for instance to Mars, will expose astronauts to extended periods of space radiation and mechanical unloading both of which may induce significant muscle and bone loss [1–3]. Together, osteopenia and sarcopenia following unloading present an increased risk of injury and morbidity [4–7]. Sources of space radiation vary with some originating from solar particle events (SPE) resulting from mass ejections from the sun known as solar flares in addition to background radiation referred to as galactic cosmic radiation (GCR) [1, 8, 9]. Space radiation from SPEs and GCR is composed of a complex mix of ions. Approximately 87% of GCR comes from protons, 12% from alpha particles, with less than 1% being high (H) atomic number (Z) and energy (E) particles (HZE). However, due to their high energy transfer HZE particles can contribute 41% of the radiation dose equivalent. SPEs are also predominantly composed of protons with smaller amounts of alpha and HZE particles [1, 8, 9]. Therefore, most of the space radiation dose equivalent originates from protons and HZE. In this study, we evaluated the catabolic effects associated with simulated space radiation comprised of proton radiation alone, and protons in combination with HZE radiation.

Recently, increased attention has focused on bone-muscle interactions and the potential that changes in one tissue may influence the other [10–13]. Myokines secreted from muscle, such as myostatin, negatively impact bone remodeling [14, 15]. Similarly, bone secreted factors, such as osteocalcin, can impact muscle and metabolism. Specific to unloading, Lloyd et al. [16] demonstrated an interdependent relationship between muscle and bone where muscle catabolism preceded bone loss induced by hind limb suspension (HLS) unloading.

While previous studies have examined the effects of protons or HZE on bone or muscle with unloading, this is the first study to examine the effects of protons and HZE on bone and muscle during unloading, a situation that is more similar to what would be experienced during extended space travel. Previous studies at the NASA Space Radiation Laboratory (NSRL) in Brookhaven, NY have demonstrated bone loss with low dose (50 cGy) HZE (^56^FE) radiation for both ground control and HLS mice, but did not examine muscle [17, 18]. Furthermore, many interventions have been examined to attenuate either catabolic bone or muscle losses [19, 20], but not both. This knowledge gap highlights a need to simultaneously investigate both tissues to better understand their relationship during unloading. To examine this issue we evaluated changes in bone and skeletal muscle in mice exposed to HLS unloading, in addition to proton and HZE (^16^O) radiation. HLS is a well validated ground based rodent model for mechanical unloading. Developed by Morey-Holten and Globus [21], HLS sufficiently unloads the hind limbs to consistently induce bone and muscle loss, while also producing a cephalic fluid shift seen in astronauts and bed restricted patients. Amongst other models of unloading [22, 23], HLS is currently the most effective and practical method for ground based rodent research. Interestingly, previous studies from our lab suggest that mechanical loading attenuates radiation induced bone loss, suggesting an inverse relationship between mechanical load and susceptibility to radiation-induced bone loss [24]. Therefore, we hypothesized that decreased mechanical load, as in the microgravity of space, would lead to increased susceptibility to space radiation-induced bone and muscle loss.

## Materials and methods

### Animal procedures

Wild type (WT) male C57BL/6J mice (Jackson Laboratories, Bar Harbor, ME, USA) were used for all experimental groups. All animals used were delivered directly from Jackson Laboratories to Brookhaven National Lab, and housed together until the start of the study. All mice used were approximately 112 days (16 weeks) old ± 3 days and skeletally mature at day 0 (start of experiment). GC and HLS mice were housed in standard polycarbonate enclosures modified for HLS (2 mice/cage), with the room temperature at 25°C and on a 12 hour light/dark cycle. Standard rodent 2018 Tekland Global 18% protein rodent diet (Harlan Laboratories Inc., Indianapolis, IN, USA) was provided ad libitum throughout the study. Mice were acclimated to the room used for the study for 2 weeks prior to study the start of the study. Mice included in radiation groups were transferred on day 3 to the NSRL building for 4 hours during which they were exposed to radiation. They were returned to the general housing facility with control mice for the remainder of the study. Mice were randomly assigned to each experimental group. At the time the experiment was started, body weight did not differ between any of the six groups (data not shown). All procedures followed NIH guidelines for experimental animals, and were approved by the Penn State Institutional Animal Care and Use Committee (Protocol # 46510).

### Simulated space radiation

Radiation administration was performed at the Brookhaven National Laboratory (Long Island, NY) through the NSRL. Proton radiation dose was 50 cGy, administered at 2 cGy/min (~25 minutes) at 150 MeV energy. Groups receiving HZE were given an additional 10 cGy ^16^O HZE radiation administered at 1 cGy/min (10 minutes) at 600 MeV energy. Beam size was 60cm x 60cm, which accommodated a custom HLS rack used for the radiation sequence. Excluding the outer frame, which was not used for irradiation, beam uniformity was 3% or better, with an overall dose uncertainty of 3.6% throughout the radiation field [25]. The local tissue field approach was used, which delivers the mix of ions at a dose profile similar to what an astronaut would incur within a space vehicle. This approach, along with the alternative external field approach have been described previously [25, 26]. All calibration and beam operation was carried out by NSRL scientists and technicians.

### Mice hind limb suspension procedure

We used a modified model of HLS originally described by Morey-Holton and Globus [21], and previously reported by our lab [16, 27]. Mice were anesthetized with isoflurane (2% + oxygen) and two tape strips were applied to the tail. This tape was attached to a swivel and string used to suspend the mouse to a metal bar across the top of the cage. Strings were adjusted to support the mouse at 30° of elevation, which is adequate for consistent HLS unloading while avoiding unnecessary strain on the animal [21]. Mice were suspended in cages as pairs; however, tethering to the suspension apparatus prevented physical contact. Control mice were placed in a similar housing environment as pairs (littermates) but were not suspended. Mice were inspected at least twice daily. All mice received urethral cleaning twice per day using saline and alcohol wipes to prevent plugs from forming, a common observation in hind limb suspended male mice [27].

### Micro-computed tomography

Right femurs were scanned using a Scanco vivaCT 40 microCT (Scanco Medical AG, Bruttsellen, Switzerland) at the conclusion of the study. Scans were performed on bones that had been wrapped in phosphate buffered saline soaked gauze and placed in 1.5ml tubes and frozen at -80°C following removal from the animal. Trabecular sections from distal femur were evaluated over a 72 slice region, consistent with previous reports [16, 27, 28]. Cortical sections from the mid-shaft of femur were evaluated over a 22 slice region. Settings were 55 KVp, 145 μa, 200ms integration time. Image reconstruction was 2048 x 2048 x 76 isotropic voxels 10.5 μm wide. Images were Gaussian filtered (sigma = 1.5, support = 2) and 27.5% threshold was used to remove soft tissue. Trabecular 72 slice regions were manually segmented, while cortical 22 slice region outlines (periosteal and endosteal boundaries) were segmented with a semi-automated edge detecting sequence. Data were analyzed in a blinded manner according to previous guidelines [29]. Trabecular parameters included bone volume percentage (Tb. BV/TV), number (Tb.N), thickness (Tb.Th), separation (Tb.Sp), connectivity density (Conn. D), and tissue mineral density (TMD). Cortical parameters included total bone volume (TV), total bone volume (BV), bone volume fraction percentage (Ct. BV/TV), thickness (Ct. Th), porosity (Ct. Po), and bone mineral density (Ct. BMD).

### Biomechanical testing

Bones stored at −80°C after microCT scanning were thawed and mechanically tested to failure via three-point bending using a Bose testing apparatus (Bose Corporation, ElectroForce Systems Group, Eden Prairie, MN, USA) as previously described [16].

### Protein synthesis

In vivo muscle protein synthesis was assessed using the SUnSET method and an antibody against puromycin (Kerafast, Boston, MA) described previously [30, 31]. All animals received an intraperitoneal injection of 0.04 μmol/g body weight of puromycin dissolved in sterile saline 30 min prior to tissue collection. Muscles (gastrocnemius and quadriceps) were subsequently excised, frozen with liquid nitrogen-cooled clamps, weighed, and stored at -80°C. Western blotting procedures were performed to visualize puromycin incorporation [30, 31].

### RNA extraction and real-time quantitative PCR

Total RNA was extracted using Tri-reagent (Molecular Research Center, Inc.; Cincinnati, OH) and an RNeasy mini-kit (Qiagen; Valencia, CA). Muscle was homogenized in Tri-reagent followed by phenol/chloroform extraction according to the manufacturer’s instructions. An equal volume of 70% ethanol was then added to the aqueous phase and the mixture loaded on a Qiagen mini-spin column. A Qiagen mini-kit protocol was followed from this point, including on-column DNase I treatment to remove residual DNA contamination. RNA was eluted from the column with RNase-free water and an aliquot used for quantitation (NanoDrop 2000, Thermo Fischer Scientific, Waltham, MA). Quality of the RNA was analyzed on a 1% agarose gel. Total RNA (1 μg) was reversed transcribed to cDNA using superscript III reverse transcriptase (Invitrogen, Carlsbad, CA) in a total reaction volume of 20 μl following instructions from the manufacturer. RT-qPCR was performed on 1–2 μl of the reverse transcribed reaction mix in a StepOnePlus system using TaqMan gene expression assays (Applied Biosystems, Foster City, CA) for the following atrogin-1, muscle RING-finger 1 (MuRF1) and insulin-like growth factor (IGF)-I, as described previously [32]. Following validation, data were expressed relative to GAPDH or rp132 expression. The comparative quantitation method 2^-ΔΔCt^ was used in presenting gene expression in reference to controls.

### Western blot analysis

Muscle was homogenized in ice-cold buffer (in mM): 20 HEPES (pH 7.4), 2 EGTA, 0.2 EDTA, 100 KCl, 50 *β*-glycerophosphate, 50 NaF, 0.5 sodium orthovanadate, 1 Benzamidine, 0.1 PMSF and 1 DTT. The protein concentration was quantified and equal amounts of protein were subjected to SDS-PAGE. Western analysis was performed for phosphorylated 4E-BP1 (Cell Signaling Technology (CST); Boston, MA), total 4E-BP1, (Bethyl Laboratories; Montgomery, TX), phosphorylated S6K1 (Thr 389) (CST), and total S6K1 (Santa Cruz Biotechnologies; Dallas, TX), Tubulin (CST), and puromycin (described above). Blots were developed with enhanced chemiluminescence reagents (Supersignal Pico, Pierce Chemical; Rockford, IL). Dried blots were exposed to x-ray film to achieve a signal within the linear range, scanned, and quantified using Scion Image 3b software (Scion Corp; Frederick, MD). Samples from all six experimental groups were run on the same gel and phosphorylated levels are expressed relative to total amounts of the respective protein.

### Statistical analysis

Data are shown as mean ± standard error. Data were analyzed with GraphPad Prism (La Jolla, CA) using two-way ANOVA, and one-way ANOVA with Student-Neuman-Keuls post hoc analysis when evaluations within a group were compared. Statistical significance was set at p<0.05.

## Results

### Trabecular bone in ground based control mice

**Fig 1:**
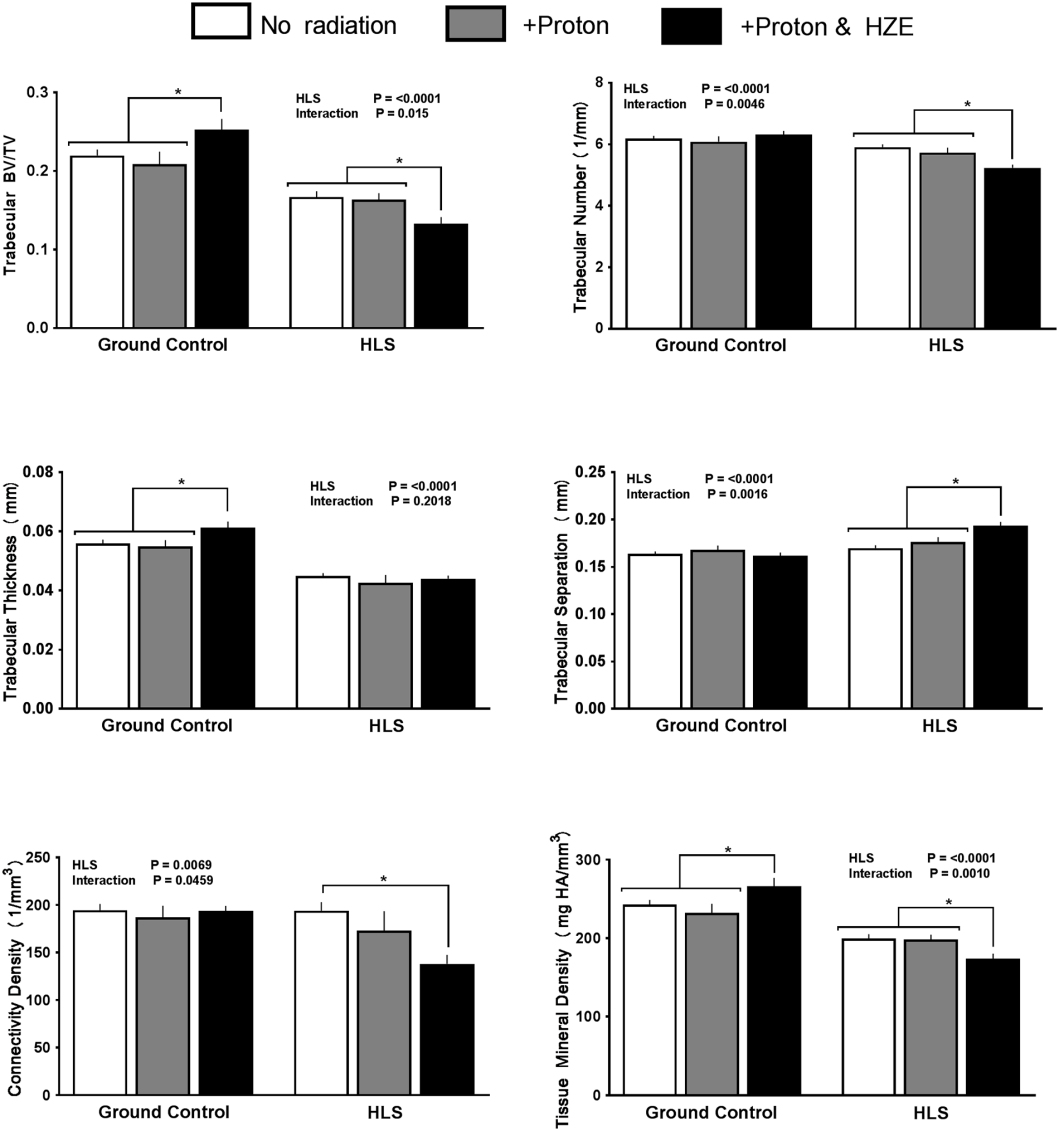
Effect of HLS and radiation on trabecular parameters measured with MicroCT scans at distal femur. Measures are shown as absolute values at conclusion of study for trabecular bone volume fraction (Tb.BVTV), trabecular thickness (Tb.Th), trabecular separation (Tb.Sp), trabecular number (Tb.N), trabecular connectivity density (Conn.Dens) and trabecular tissue mineral density (Tb.TMD). Values are means ± SEM; n = 14–18 bones/group. Values with line and asterisk indications are significantly different (p<0.05).

**Fig 2:**
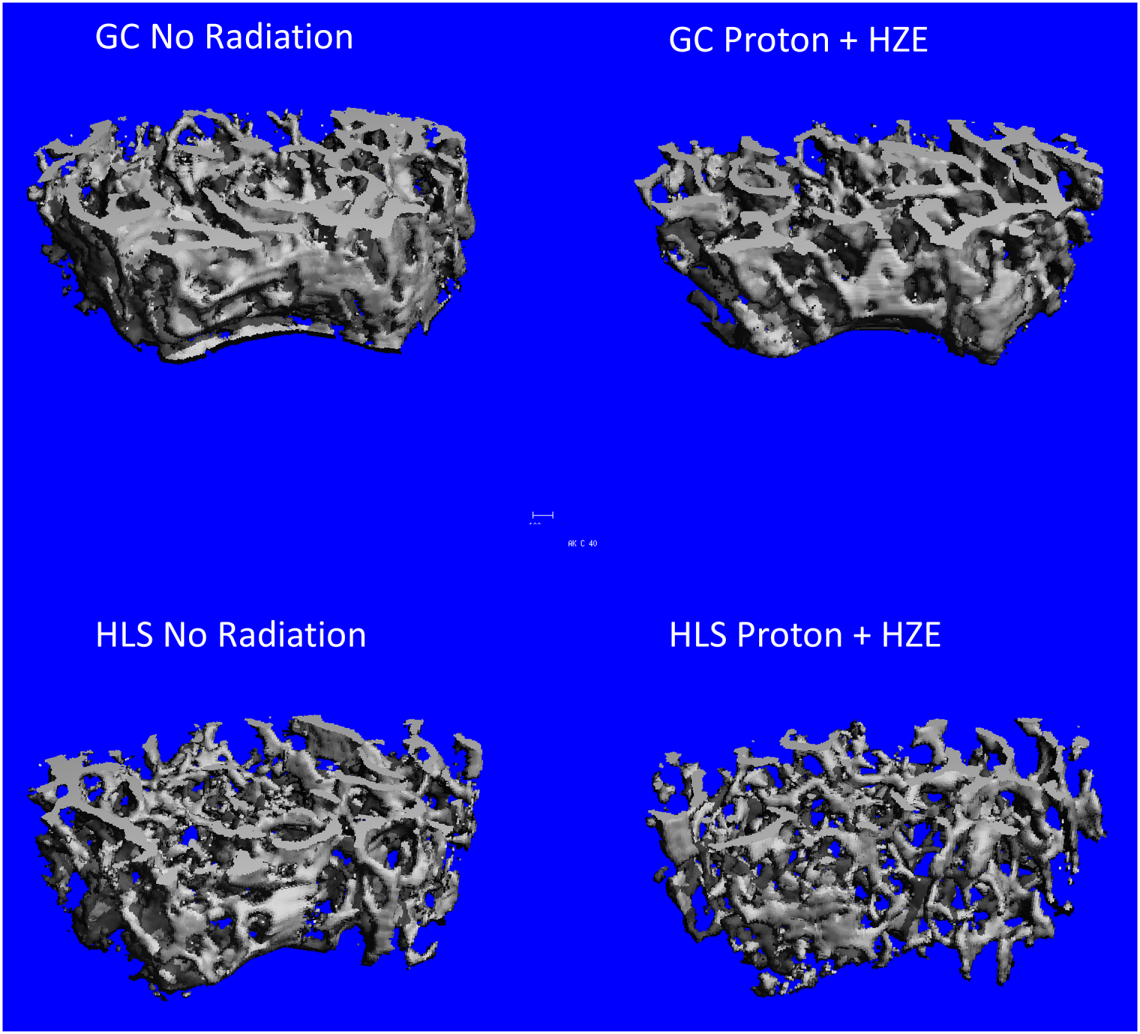
Representative images of trabecular samples from GC mice (top) and HLS mice (bottom) on day 14. Images represent a 72 slice region (756 μm) of distal femur.

Many of the endpoints related to trabecular bone did not differ among the three groups of unloaded mice, regardless of radiation treatment. However, some parameters including trabecular bone volume fraction (BV/TV)(+12% difference), trabecular thickness (+11% difference), and trabecular tissue mineral density (+9% difference) showed an unexpected anabolic response for the proton+HZE group compared to GC mice receiving no radiation (Fig 1). Proton radiation alone did not have an effect on any trabecular outcome compared to the non-radiated GC group. Visual representation generated by microCT analysis for trabecular bone samples can be seen in Fig 2.

### Trabecular bone in HLS unloaded mice

At the conclusion of the 14-day HLS period, unloading had increased trabecular bone loss as evidenced by the reduction in bone volume fraction (-25%), trabecular thickness (-20%), and tissue mineral density (-19%) (Fig 1). Other trabecular parameters did not change with HLS alone compared to GC values. In mice subjected to HLS and proton+HZE radiation, additional trabecular loss was observed for bone volume fraction (-20%), trabecular number (-12%), trabecular separation (+12%), connectivity density (-29%), and tissue mineral density (-13%) when compared to either the HLS alone or HLS+proton groups (Fig 1). HLS mice subjected to only proton radiation did not show additional loss compared to HLS alone (Fig 1).

### Cortical bone in ground control mice

**Fig 3:**
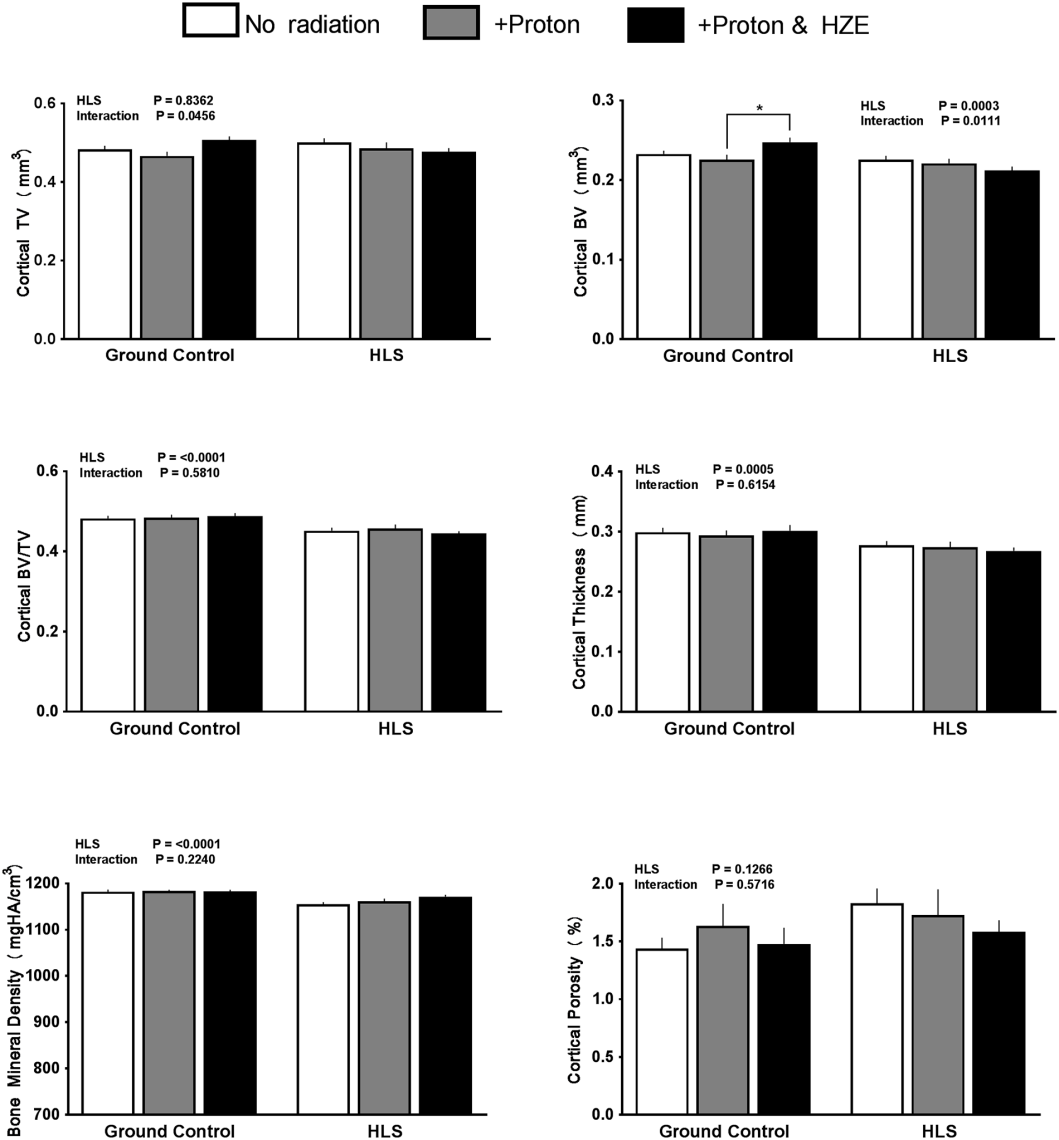
Effect of HLS and radiation on cortical parameters measured with MicroCT scans at distal femur. Measures are shown as absolute values at conclusion of study for cortical total volume, cortical bone volume, cortical bone volume fraction (Ct. BVTV), cortical thickness, cortical bone mineral density, and cortical porosity. Values are means ± SEM; n = 14–18 bones/group. Values with line and asterisk indications are significantly different (p<0.05).

In general, there were no changes to cortical bone with radiation treatment. One outcome (bone volume) showed a small anabolic shift (+6%) in the proton+HZE group (Fig 3), however this was the only change among GC groups.

### Cortical bone in HLS unloaded mice

Similar to GC groups, radiation treatment did not significantly alter cortical outcomes. Cortical BV/TV and cortical bone mineral density showed a catabolic change with HLS compared to GC (-7% and -3% respectively), but did not show additional loss associated with either type of radiation (Fig 3). In response to proton & HZE during HLS, bone mineral density actually increased compared to HLS alone and was equal to GC groups. HLS alone or HLS in conjunction with radiation treatment altered other cortical outcomes.

### Biomechanical testing

**Fig 4:**
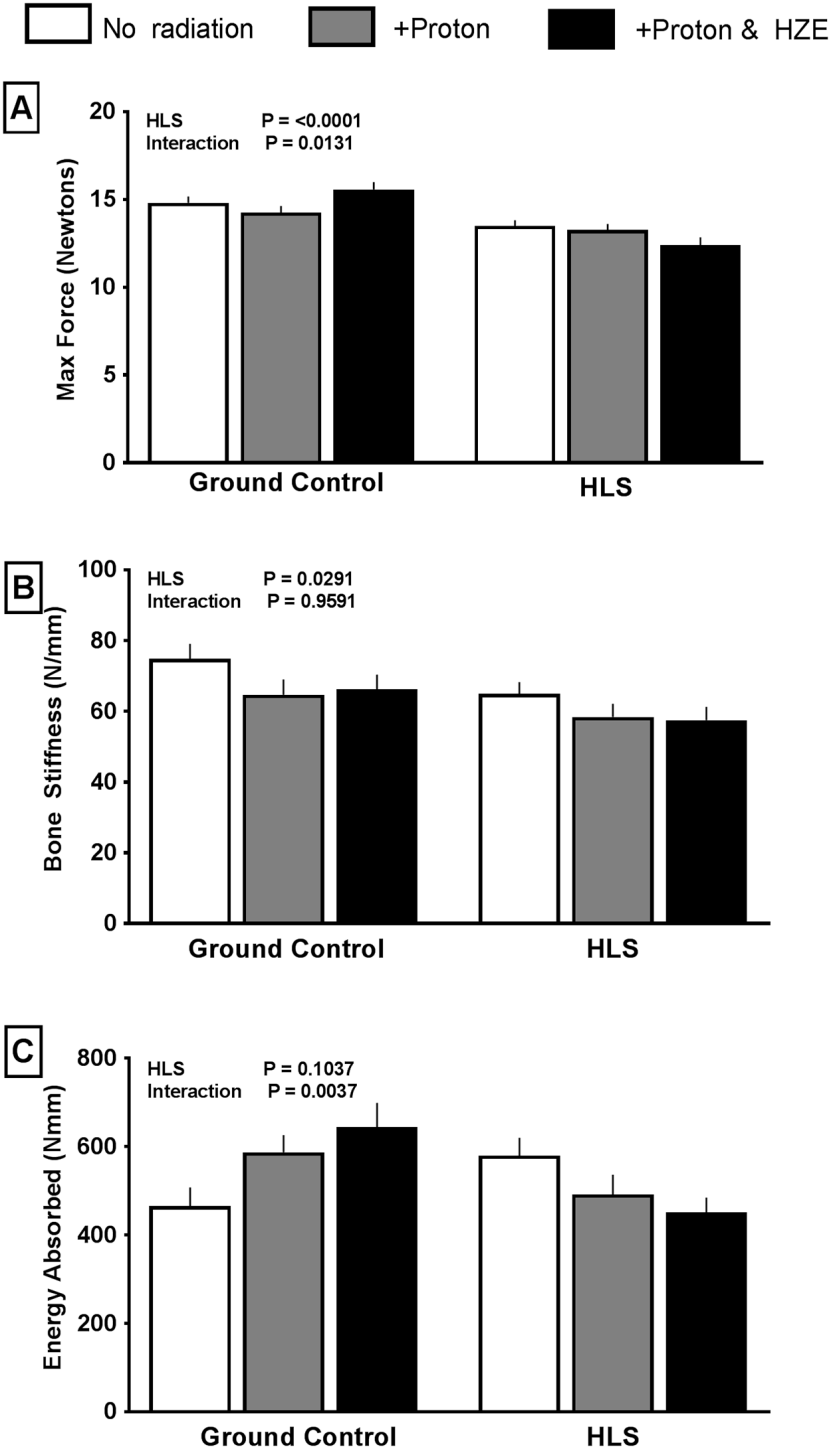
Effect of HLS and radiation on mechanical properties of femurs observed during 3 point bending until failure. Measures are shown as absolute values at conclusion of study for max force during testing (A), bone stiffness (B), and energy absorbed during testing (C). Values are means ± SEM; n = 14–18 bones/group. Values with line and asterisk indications are significantly different (p<0.05).

Femurs that underwent 3 point mechanical testing showed a decrease in max force (-10%) during break in the HLS groups compared to the GC groups; however, radiation did not exacerbate the HLS-induced decrease in breaking strength and did not have on effect on GC groups (Fig 4A). No difference was observed in total energy absorbed during break across any group (Fig 4C).

### Muscle weight and protein metabolism

**Fig 5:**
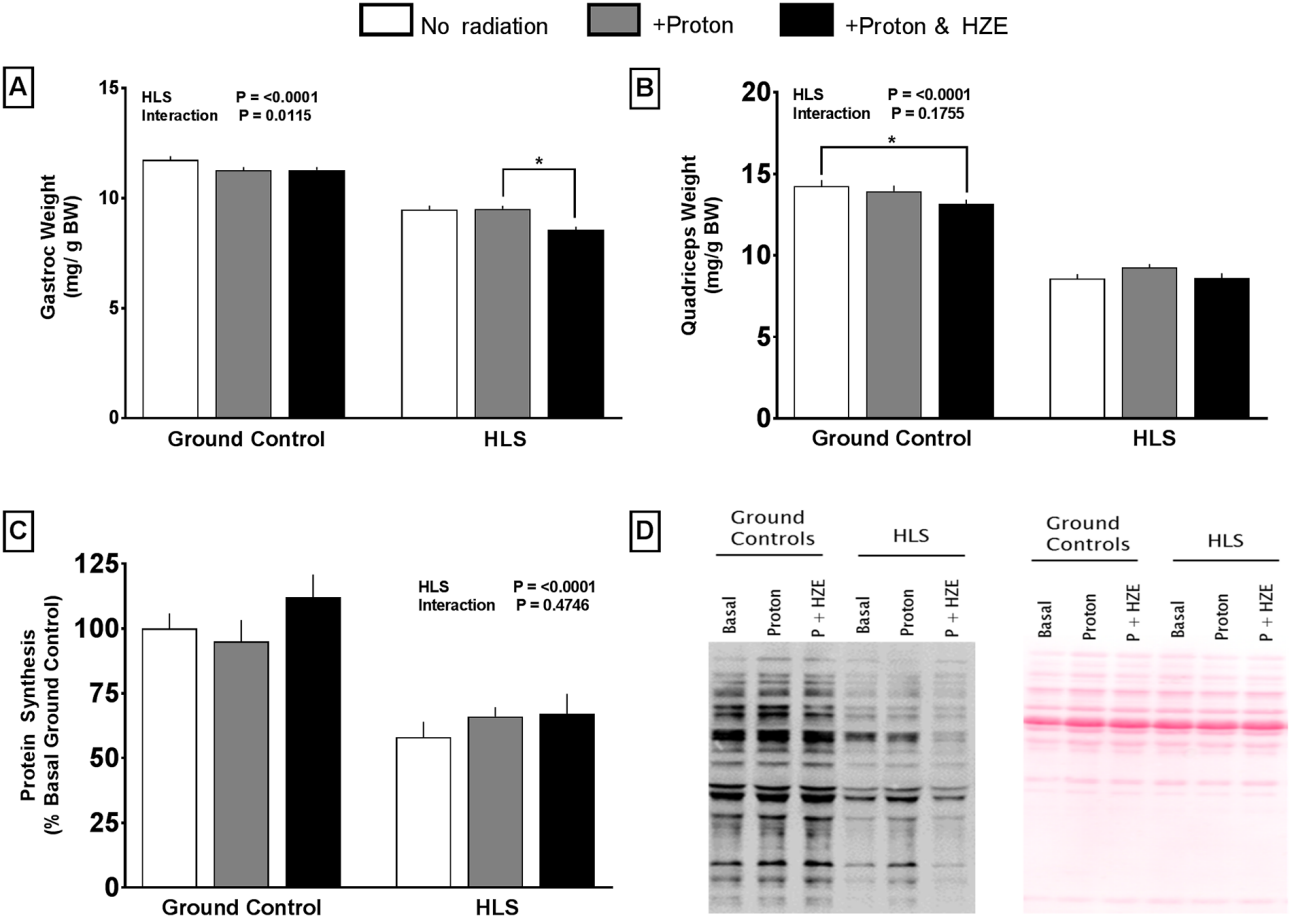
Effect of HLS and radiation on wet muscle weight of gastrocnemius (A), quadriceps (B), and protein synthesis (C) measured with the SUnSET method. Representative Western blot of Puromycin incorporation into muscle protein for each of the six experimental groups (D left), and representative Ponceau stain demonstrating equal protein loading per lane (D right). Measures are absolute values normalized to body weight at conclusion of study (GC in protein synthesis). Values are means ± SEM; n = 14-18/group (p<0.05).

For gastrocnemius weight, there was no difference in wet muscle weight between the three GC groups (Fig 5A). In contrast, the wet weight of the quadriceps was less (-8%) in the proton+HZE radiation group compared to the other two GC groups (Fig 5B). For mice receiving only proton radiation, muscle weights did not differ from non-radiated groups. Protein synthesis determined in vivo (normalized to GC-no radiation) did not show a difference between the three GC groups (Fig 5C).

Gastrocnemius weight was decreased (-19%) in HLS mice vs GC (Fig 5A). While proton radiation alone did not affect HLS muscle weight, proton+HZE radiation resulted in an additional loss in gastrocnemius weight (-9%) compared to HLS alone (Fig 5A). In the quadriceps, HLS reduced weight (-39%) compared to GC. In contrast to gastrocnemius data, the combination of HLS+proton+HZE did not exacerbate muscle loss (Fig 5B). Protein synthesis in the gastrocnemius was consistently lower in all three HLS groups compared to GC groups (~40%), but was not affected by radiation (Fig 5C).

**Fig 6:**
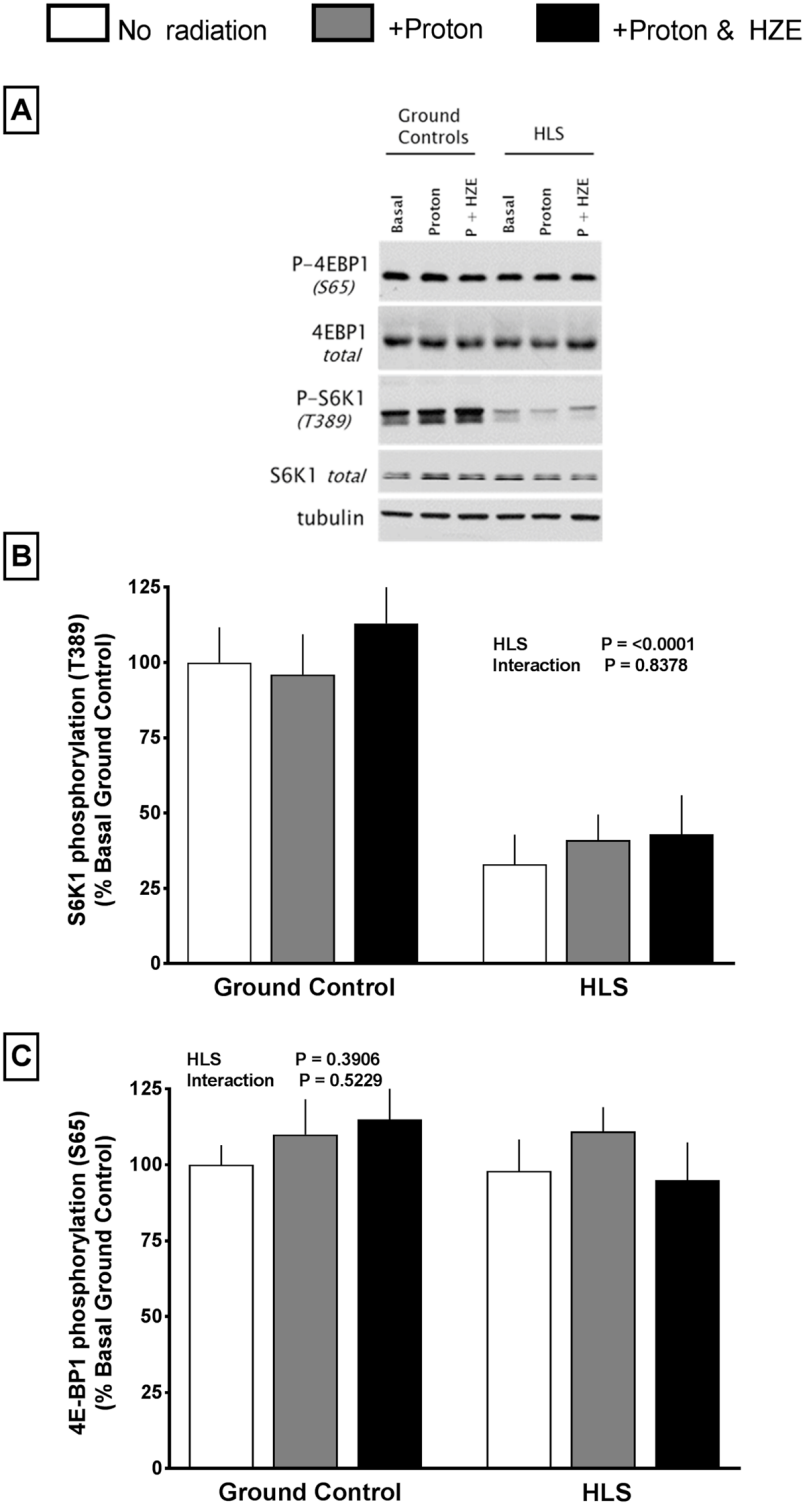
Effect of HLS and radiation on phosphorylation of mTOR downstream regulators 4EBP1 and S6K1 measured via Western blot analysis. Top panel (A) is a representative Western blot for phosphorylated and total S6K1 (B) and 4E-BP1 (C) in muscle from each of the six experimental groups; tubulin is also included to demonstrate equal protein loading all groups. All samples were run on the same gel. Bar graphs represent average values at conclusion of study and are normalized to GC group. Values are means ± SEM; n = 14-18/group, (p<0.05).

The mammalian target of rapamycin complex 1 (mTORC1) regulates muscle protein synthesis at least in part by phosphorylation of 4E-BP1 and S6K1 [33]. There was no difference in S6K1 phosphorylation (Thr389) between the three ground control groups (Fig 6A); however, HLS did decrease (~65%) S6K1 phosphorylation (Fig 6A). Radiation did not differentially affect S6K1phosphorylation in HLS groups. In contrast, the phosphorylation of 4E-BP1 on Ser65 in the gastrocnemius did not differ among the 6 groups (Fig 6B). These changes in phosphorylation were independent of a change in the total amount of either 4EBP1 or S6K1.

**Fig 7:**
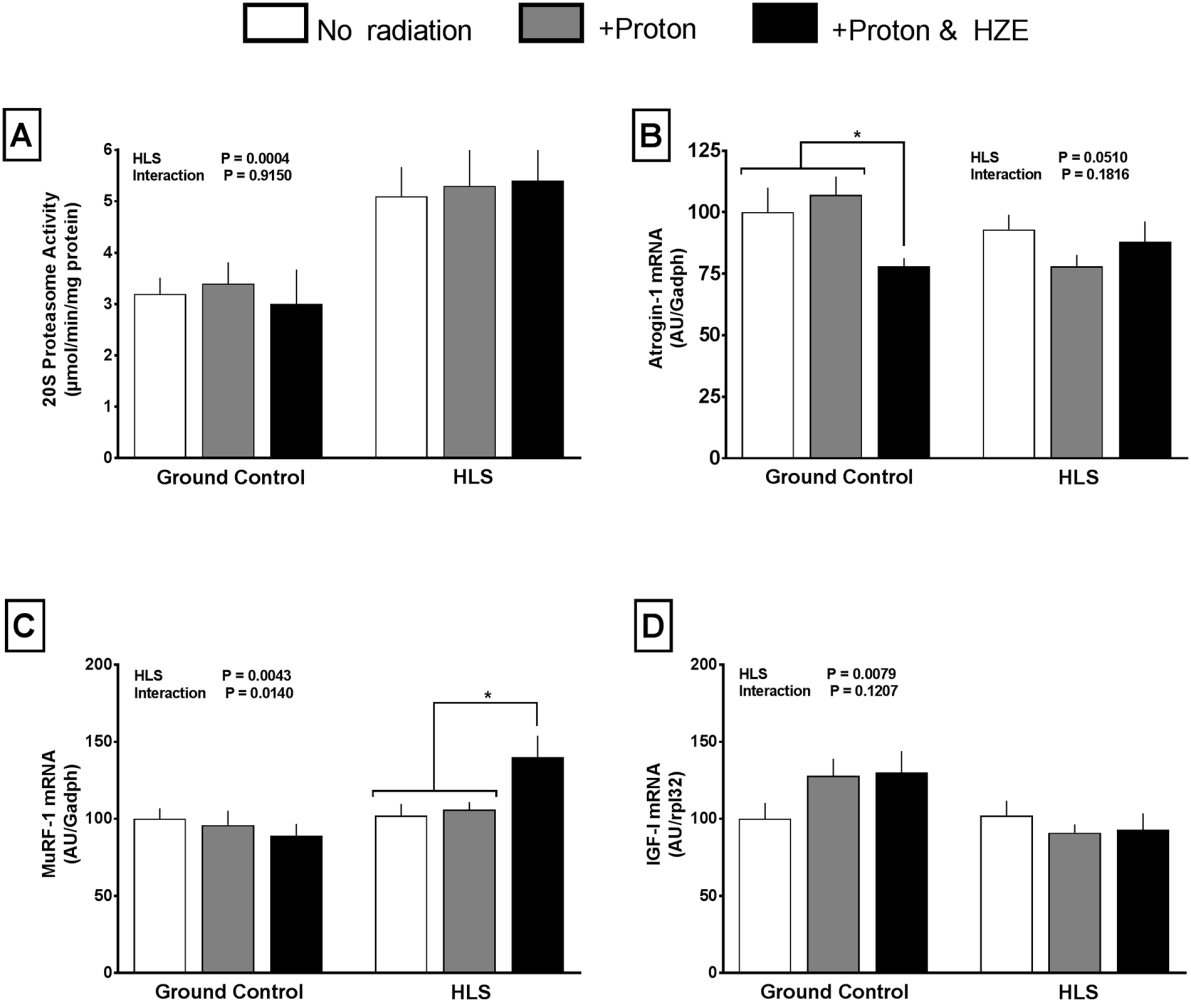
Effect of HLS and radiation on 20S proteasome(A), atrogin-1 (B), MuRF-1 (C), and insulin growth factor (D) expression. Measures are absolute values at conclusion of study. Values are means ± SEM; n = 14-18/group, Values with line and asterisk indications are significantly different (p<0.05).

The activity of 20S proteasome was increased (40%) in HLS groups compared to GC groups, but neither the GC nor HLS groups were affected by radiation (Fig 7A). Two muscle-specific E3 ubiquitin ligases that regulate protein degradation, atrogin-1 and MuRF1, showed little change between groups. A decrease in atrogin-1 activity (-22%) was observed in the GC proton+HZE group, as well as the HLS proton (-22%) group relative to the GC no radiation group (Fig 7B). MuRF-1 mRNA was increased in the HLS proton+HZE group (+40%), and was not changed in others groups relative to the GC no radiation group (Fig 7C). IGF-I plays an important endocrine and autocrine role as a positive regulator of muscle mass by increasing protein synthesis and decreasing protein degradation [33]. IGF-I mRNA content also did not differ between the six experimental groups for gastrocnemius (Fig 7D).

## Discussion

We found that trabecular osteopenia developed in HLS mice during the two-week duration of the experiment consistent with previous findings regarding bone loss during unloading [16, 27]. This unloading-induced decrease was in addition to age-related changes that also occur in C57BL/6J mice of this age [16, 27, 34, 35]. In contrast, with one exception, there was little evidence of cortical bone loss associated with HLS. These data suggest a differential sensitivity of cortical and trabecular bone to the catabolic effects of HLS, and are consistent with previous studies demonstrating that 2 weeks of unloading is not sufficiently long to significantly change cortical bone. This is due in part to the slower turnover of cortical bone. We opted for a 2 week unloading period based on our previous study [16] showing significant changes for both bone and muscle relative to baseline at this time point. The lack of cortical bone loss may also be explained by previous studies that indicated cortical bone parameters in BL/6 mice continue to increase until six months of age [34, 35]. In addition, development of osteopenia in control mice independent of unloading during longer duration experiments (i.e. aging) may mask the effect of unloading. Despite little change in cortical outcomes, there was a decrease in max break force across HLS groups during 3 point mechanical testing; however, max break force was not affected by radiation. Other mechanical data showed a decrease in stiffness with HLS and radiation treatment compared to GC alone, and no change in energy absorbed during mechanical testing. At 4 months, the age of the mice in this study, we expected to see an age-dependent decline in trabecular bone endpoints [16, 34, 35], along with muscle loss, associated with HLS.

While trabecular osteopenia developed with HLS, several endpoints showed additional loss with radiation. Our data indicate that the proton only dose (50cGy) did not have a detectable effect on bone outcomes. However, the addition of HLS in conjunction with (10cGy) HZE radiation, which has a significantly higher energy and represents a large portion of the dose equivalent, caused additional bone loss. Interestingly, proton+HZE radiation caused an anabolic response for some trabecular outcomes in ground control mice; the mechanism for these results is not understood. Proton+HZE radiation is a more accurate representation of galactic cosmic radiation experienced by astronauts than is proton radiation alone [8, 9], and the 60cGy total dose (0.6 Gy) is representative of radiation exposure during a mission duration of approximately 400 days [36]. Extended time outside a shielded vehicle may significantly increase (up to 4x) the dose rate, while extended solar particle events may also increase the cumulative dose [36]. Therefore, it is reasonable to conclude based on our findings that there would be significant bone complications with extended space travel missions resulting from unloading, which is further exacerbated by space radiation exposure.

Previous studies have reported that the quadriceps appears to be less sensitive than the gastrocnemius muscle to unloading-induced atrophy [16, 37–39]; however, we observed greater loss in the quadriceps compared to the gastrocnemius weight in groups of mice exposed to HLS. The reduction in muscle weight appears to result, at least in part, from a decrease in global protein synthesis. The activity of mTORC1 is a key regulator of muscle protein synthesis [33] and our data show that the decrease in protein synthesis was associated with concomitant reduction in the phosphorylation of the mTORC1 substrate S6K1. Phosphorylation of S6K1 and 4E-BP1, two downstream surrogate markers of mTOR, differed at day 14. S6K1 phosphorylation was decreased in all HLS groups, while 4E-BP1 phosphorylation did not differ between groups at day 14. This dichotomy was consistent with previous reports that 4E-BP1 phosphorylation was not significantly altered until day 21 of unloading [16]. The disconnect between 4E-BP1 phosphorylation and protein synthesis (which is significantly decreased at times when 4E-BP1 is not) is not fully understood, but is consistent with our understanding that many factors affect protein synthesis. Thus, it is important to independently evaluate protein synthesis and its signal transduction pathway.

Proteolysis also regulates muscle protein balance in part via alterations in the ubiquitin proteasome pathway [40]. In this regard, 20S proteasome activity was increased by HLS alone compared to GC values, and was not exacerbated by the concomitant exposure to radiation. This HLS-induced increase in proteasome activity appeared independent of changes in the mRNA content of the atrogenes atrogin-1 and MuRF1, that are up-regulated in a number of catabolic conditions [40, 41]. MuRF1 did show a significant increase in the HLS proton+HZE, which may partially explain the decrease in muscle weight in the same group despite no change in global protein synthesis.

Our data are consistent with a previous study that showed no effect of space-like radiation (albeit a different type, x-ray) in combination with HLS on muscle size at much higher doses than used in this study [42]. This, along with our data suggests muscle is less susceptible than bone to radiation-induced catabolism, although still clearly catabolic in the unloading environment. Another potential consideration for the preservation of muscle is the developing link between osteocalcin and other metabolic systems [43, 44]. Osteocalcin is derived from osteoblasts and embedded in bone matrix, and is subsequently released into the bloodstream during bone resorption and osteoclast activity. Undercarboxylated osteocalcin can affect fat metabolism, insulin sensitivity, and increase testosterone production via Leydig cell stimulation [43, 44]. Mice being irradiated would have increased osteoclast activity, increasing the circulating osteocalcin and thus potentially increasing the anabolic hormone testosterone; increased testosterone could have a protective effect on muscle. Neither serum osteocalcin or testosterone concentrations were measured in the present study, but represent an avenue for future research as an interesting potential bone-muscle interaction.

In summary, our results demonstrate for the first time, that exposure to specific simulated space radiation (60cGy of proton+HZE) sensitizes bone to the catabolic effects of simulated microgravity resulting in additional bone loss. Interestingly, this was not the case for unloading-induced muscle loss. As radiation exposure alone did not affect either muscle or bone, countermeasures to mitigate unloading-induced bone and muscle loss may also mitigate the catabolic effects of exposure to space radiation.
